# Sympathetic skin response (SSR) in pediatric Guillain–Barré syndrome

**DOI:** 10.3389/fneur.2023.1177394

**Published:** 2023-06-09

**Authors:** Mei Jin, Jing Liu, Kang Liu, Libo Zhao, Ziwei Zhao, Suzhen Sun

**Affiliations:** ^1^The Children Hospital of Hebei Province, Shijiazhuang, Hebei, China; ^2^The Key Laboratory of Pediatric Epilepsy and Neurological Disorders of Hebei Province, Shijiazhuang, Hebei, China

**Keywords:** Guillain-Barré syndrome, children, sympathetic skin response, autonomic dysfunction, short-term and long-term prognosis

## Abstract

**Introduction:**

In the present study, sympathetic skin response (SSR) characteristics were explored in children with Guillain–Barré syndrome (GBS) and the value of early diagnosis and prognostic evaluation in GBS complicated by autonomic dysfunction (AD) was evaluated.

**Methods:**

A total of 25 children with GBS and 30 healthy controls (HCs) were enrolled in this prospective study. SSR findings for the two groups were compared. SSR and nerve conduction study (NCS) results were compared among patients with GBS, and differences in clinical characteristics between the groups with abnormal and normal SSR were analyzed.

**Results:**

Within the GBS group, six patients (24%) required mechanical ventilation, 17 patients (66.7%) had AD, 18 patients (72%) had an abnormal SSR, and 13 patients (52%) had AD combined with SSR abnormalities. There was a statistically significant difference in SSR latency in the lower limbs between the GBS group and HCs (*P* < 0.05). There was no statistically significant difference between SSR and NCS results in the acute phase of GBS (*P* > 0.05), and there was no significant difference in the rate of AD or in Hughes functional grade at nadir between the groups with abnormal and normal SSR (*P*>0.05). However, there was a statistically significant difference between the results of SSR and NCS tests during the recovery phase (*P* = 0.003). Abnormal SSR mainly occurred in cases of the acute inflammatory demyelinating polyradiculoneuropathy (AIDP) subtype. In addition, SSR was abnormal in all pediatric GBS patients with poor prognosis 1 month after onset of symptoms.

**Conclusion:**

Two-thirds of the children with GBS have AD. SSR could be used for early diagnosis and follow-up of GBS and may also be helpful in evaluating disease severity and short-term prognosis.

## 1. Introduction

Guillain–Barré syndrome (GBS) is the most common cause of acute flaccid paralysis in children (1). The incidence of GBS in China is approximately 0.698 per 100,000 person-years, and the incidence is lower in children than in adults (0.233 in children vs. 0.829 in adults) (2). Acute inflammatory demyelinating polyradiculoneuropathy (AIDP) and acute motor axonal neuropathy (AMAN) are the most common subtypes, accounting for ~69–90% and 5–70% of GBS cases, respectively (3). The diagnosis and classification of GBS in children is challenging, especially in younger children, where this is more dependent on auxiliary examinations. Therefore, electrophysiological examination is of great significance for the early diagnosis, classification, and prognostic assessment of GBS.

Two-thirds of patients with GBS have autonomic dysfunction (AD) (4), such as tachycardia, hypertension, or abnormal sweat secretion. Some studies have shown that AD is an independent risk factor for poor prognosis in patients with GBS and can even lead to death (5, 6). Therefore, we should attach greater importance to early detection of and intervention for AD in patients with GBS. The sympathetic skin response (SSR) can be used to detect the function of sympathetic nerve fibers by recording the skin-sweating response via surface electrodes; this is a relatively objective electrophysiological marker for analysis of autonomic functions (7). SSR has been used in the diagnosis, differential diagnosis, and assessment of prognosis in many neurological diseases, such as diabetic peripheral neuropathy (8), amyotrophic lateral sclerosis (9), and multiple sclerosis (10). The present study aimed to explore the characteristics of the SSR in children with GBS and analyze the value of early diagnosis and prognostic evaluation in GBS complicated by AD.

## 2. Materials and methods

### 2.1. Participants

We prospectively recruited GBS patients who were admitted to our neurology department between 2018 and 2022. Inclusion criteria for the GBS group were as follows: (I) patients aged ≥4 years and ≤16 years; (II) those who met level 2 of the Brighton criteria for GBS (11, 12); and (III) those who underwent at least two neuroelectrophysiological tests (within 2 weeks of onset, and between 4 and 8 weeks after onset). Patients with acute chronic inflammatory demyelinating polyradiculoneuropathy or other neuropathies (such as genetic, metabolic, or nutritional neuropathy, or neuropathy with toxic causes) were excluded. Children diagnosed with Tourette syndrome (aged ≥5 years and ≤16 years) were selected for recruitment as healthy controls (HCs). All HCs had unremarkable cranial magnetic resonance imaging findings, were not taking any drugs with anti-cholinergic effects, and presented without clinical features of nerve system damage or any other autoimmune or oncological diseases. This study was approved by the Ethics Committee of the Children's Hospital of Hebei Province.

### 2.2. Methods

#### 2.2.1. Clinical and laboratory data

The following clinical characteristics were evaluated: presence of preceding events; time from symptom onset to nadir; occurrence of facial paralysis, bulbar paralysis, autonomic dysfunction (such as tachycardia or bradycardia, hypertension or hypotension, urinary retention, or sudomotor dysfunction); requirement for mechanical ventilation; and form of treatment. Protein level and white blood cell count in the cerebrospinal fluid were also analyzed.

#### 2.2.2. GBS disability score

Each patient's degree of motor disability was assessed according to Hughes functional grading (HG: 0 = normal functional state; 1 = minor signs or symptoms, able to run; 2 = able to walk 10 m without assistance, but unable to run; 3 = able to walk with assistance; 4 = chair- or bed-bound; 5 = requiring mechanical ventilation; 6 = death) (13) at nadir and at 1 month and 6 months after onset of symptoms. Patients with HG ≥3 were classified as having a poor outcome, and patients with HG <3 were classified as having a good outcome.

#### 2.2.3. Stratification of GBS patients by disease course

Patients with GBS were stratified according to the timing of symptom presentation from disease onset (3): the acute phase was defined as the first 2 weeks after disease onset, the progressive phase was usually 2–4 weeks after disease onset, and the recovery phase was usually between 4 and 8 weeks of disease onset.

#### 2.2.4. Sympathetic skin response recording

We recorded the SSR from both the left and right palmar and plantar surfaces according to the Technical Standards of the International Federation of Clinical Neurophysiology (14). Recording electrodes were placed on the palmar and plantar regions and reference electrodes were placed on the dorsa of the hands and feet. An electrical stimulus (intensity: 20–40 mA; duration: 0.2 ms) was applied to the contralateral median nerve at the wrist. To overcome habituation and the ensuing variability, inter-stimulus intervals varied randomly within the range of 40–60 s, and the intensity of stimulation was increased progressively. At least five consecutive SSR potentials were recorded, and the mean value of all recorded SSR potentials was entered into analysis. Onset latency was measured from the beginning of the stimulus to the first continuous deflection from the baseline; N latency was measured from the stimulus to negative deflection; and P latency was measured from the stimulus to positive deflection. The latencies of SSR were analyzed rather than aplitudes, as previous studies have demonstrated that changes in the amplitude of the SSR can easily occur in response to repeated electrical stimuli during habituation, unlike latency (15). The results were considered abnormal when no response was obtained or when latency exceeded the 97.5th percentile among the HCs.

#### 2.2.5. Nerve conduction study

All procedures were performed on the upper and lower limbs. Motor nerve conduction studies (MNCSs) were performed on the median, ulnar, tibial, and peroneal nerves. F-waves were examined in the median, ulnar, and tibial nerves. Sensory nerve conduction studies of the median, ulnar, and sural nerves were performed. The parameters included distal motor latency, distal and proximal compound muscle action potentials, motor and sensory conduction velocity, and minimal F-wave latency. GBS patients were classified as having AIDP or AMAN based on the criteria described by Hughes et al. (16).

#### 2.2.6. Statistical analyses

Statistical analyses were performed using IBM SPSS Statistics version 24 (IBM Corp., Armonk, N.Y., USA). The distribution of all continuous variables was tested using the Kolmogorov–Smirnov normality test. Normally distributed continuous variables are reported in the form mean ± SD and were compared using the unpaired Student's t test. Non-normally distributed continuous variables are reported in the form of the median and interquartile range (IQR) and were compared using the Mann–Whitney U test. Categorical variables relating to clinical characteristics are reported in the form of proportions and were compared using the chi-square test or Fisher's exact probability test. Comparisons of SSR and NCS results obtained at early diagnosis of GBS and at follow-up were performed using McNemar's test. A *p*-value of 0.05 was significant.

## 3. Results

### 3.1. Clinical characteristics of pediatric GBS patients

A total of 27 children with GBS met the inclusion criteria: one patient with a pharyngeal-cervical-brachial subtype and one with a paraparetic subtype were excluded, and the remaining 25 children with GBS (14 male, 11 female) were recruited; of these 21, had AIDP and 4 had AMAN, and the median age was 6 years (IQR = 5–8).

Among the GBS group, 17 patients (66.7%) had AD, including nine with tachycardia, two with hypertension, four with urinary retention, and six with sudomotor dysfunction. Additionally, 18 patients (72%) exhibited an abnormal SSR, among whom 13 (52%) had autonomic dysfunction along with abnormal SSR findings, while five (20%) had SSR abnormalities only. In addition, six patients (24%) required mechanical ventilation, including five patients with AD and an abnormal SSR.

### 3.2. SSR findings in pediatric GBS patients and healthy controls

The HCs consisted of 30 patients (14 male and 16 female) with a median age of 11 years (IQR = 8–12). SSR could be elicited in all HCs, and the median onset latency of the SSR was 1.21 s (IQR = 1.15–1.24) in the upper limbs and 1.69 s (IQR = 1.65–1.77) in the lower limbs.

**Figure 1:**
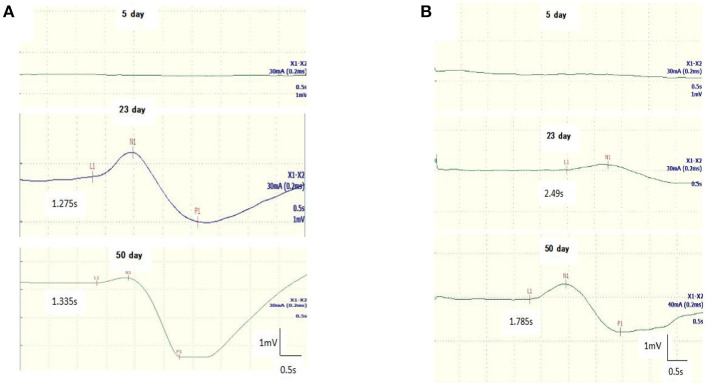
Follow-up recordings of sympathetic skin response (SSR) in the upper **(A)** and lower **(B)** limbs from a 11-year-old boy with GBS. SSR characteristics changed from the absence of a waveform on the fifth day since the onset to a prolonged latency on the 23th day, then returned to baseline on the 50th day.

**Figure 2:**
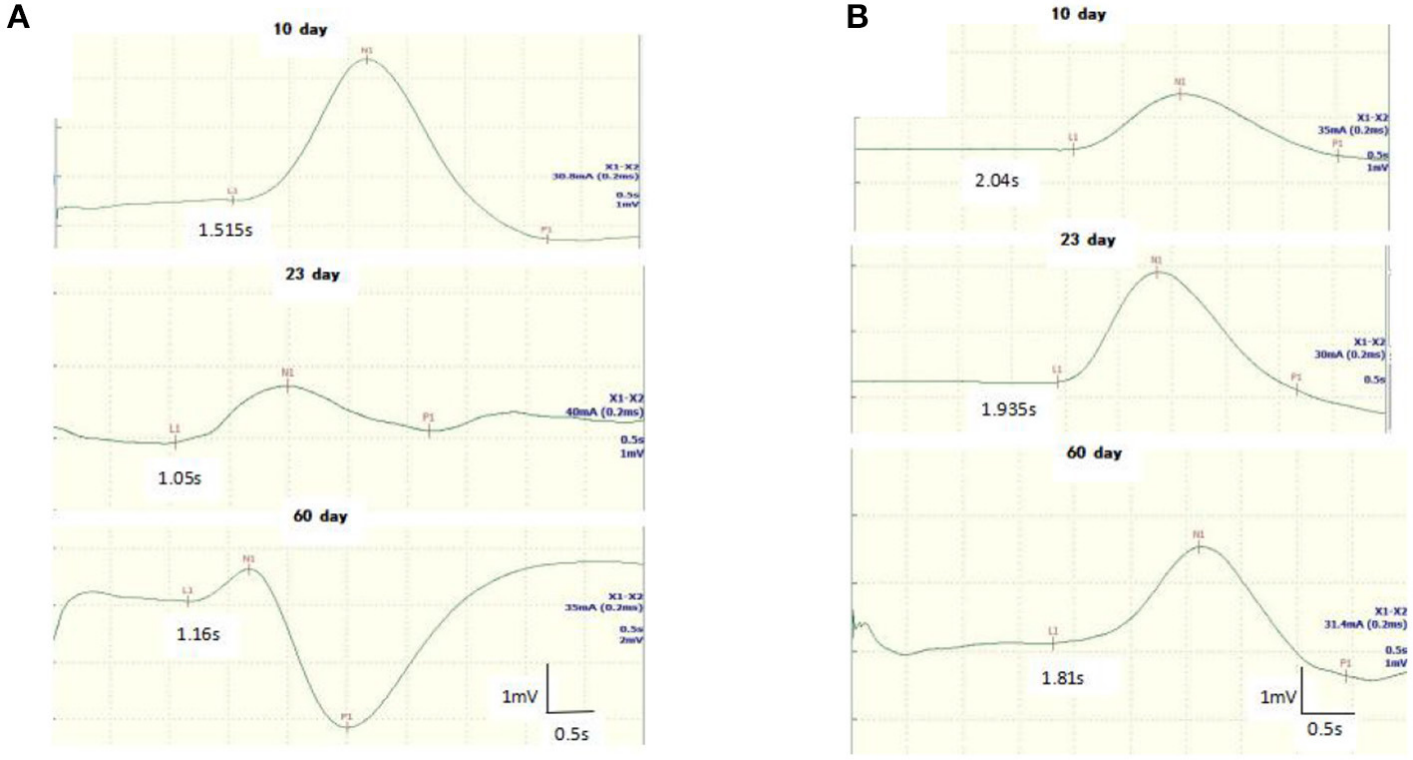
Follow-up recordings of sympathetic skin response (SSR) in the upper **(A)** and lower **(B)** limbs from a 8-year-old girl with GBS. The latency of the SSR was shortened at days 10 and 23 until it returned to baseline on the 60th day.

All GBS patients underwent neuroelectrophysiological tests in the acute phase (median time since disease onset: 9 days), and these showed absence of the SSR or prolonged latency. In these tests, eight patients (32%) had an absent SSR in the upper and lower limbs simultaneously, three patients (12%) exhibited prolonged latency in the upper and lower limbs, and seven patients (28%) exhibited prolonged latency in the lower limbs only. SSR latencies (including onset and N latency) in the lower limbs were significantly longer in the GBS group than in the HCs (*P* < 0.05). Overall, SSR abnormalities were more frequent in the GBS group than in the HCs (χ^2^ = 32.108, *P* < 0.001). Twenty-three patients also underwent neuroelectrophysiological examinations in the progressive phase (median time since disease onset: 20 days). In this phase, the SSR changed from the absence of a waveform to a response with a prolonged latency, or the prolonged latency was gradually shortened. Finally, all GBS patients underwent neuroelectrophysiological tests in the recovery phase (median time since disease onset: 50 days); in this phase, the latency of the SSR was further shortened until it returned to baseline (Figures 1, 2, Table 1).

**Table 1:** Comparison of SSR characteristics between GBS patients in the acute phase and healthy controls.

**Variable**	**GBS patients**	**HCs**	**Comparative test statistic**	***P***
Number of participants	25	30		
Age (years), median (IQR)	6 (5–8)	11 (8–12)	*Z* = 1.261	0.207
Male, *n* (%)	14 (56)	14 (46.7)	*χ^2^* = 475	0.491
Body mass index (kg/m^2^), mean ± SD	17.4 ± 2.3	18.5 ± 2.4	*T* = 0.555	0.584
**SSR (palm)**				
Onset latency (s), median (IQR)	1.26 (1.17–1.31)	1.21 (1.15–1.24)	*Z* =1.94	0.052
N latency (s), median (IQR)	1.85 (1.77–1.86)	1.85 (1.78–1.92)	*Z* = 0.401	0.688
P latency (s), median (IQR)	2.78 (2.65–2.9)	2.88 (2.73–2.96)	*Z* = 0.768	0.433
**SSR (sole)**				
Onset latency (s), median (IQR)	1.86 (1.69–2.11)	1.69 (1.65–1.77)	*Z* = 2.915	0.004
N latency (s), median (IQR)	2.7 (2.55–2.9)	2.4 (2.38–2.58)	*Z* = 3.411	0.001
P latency (s), median (IQR)	3.5 (2.85–3.6)	3.17 (2.93–3.41)	*Z* = 1.376	0.169

SSR, sympathetic skin response; HCs, healthy controls; Z, rank sum test statistic.

In the acute phase, 18 patients (72%) had an abnormal SSR and 16 patients (64%) had NCS abnormalities; the SSR abnormalities did not differ statistically from the NCS abnormalities among the GBS patients (*P* = 0.727). In addition, there were six patients (24%) with abnormal SSR and 17 patients (68%) with abnormal NCS findings in the recovery phase, and recovery of SSR occurred earlier than that of NCS during the follow-up period in GBS (*P* = 0.003) (Table 2).

**Table 2:** Comparison of NCS and SSR findings in children with GBS.

		**Acute phase SSR**		**Recovery phase SSR**	
		**Positive**	**Negative**	**Positive**	**Negative**
NCS	Positive	13	3	5	12
	Negative	5	4	1	7
McNemar test *P*-value		0.727		0.003	

SSR, sympathetic skin response; NCS, nerve conduction study.

### 3.3. Comparison of clinical data between the abnormal and normal SSR groups

No significant differences were noted between the abnormal and normal SSR groups in terms of neurological symptoms, autonomic dysfunction, requirement for mechanical ventilation, or Hughes grade at nadir, 1 month, or 6 months after symptom onset (*P* > 0.05). Neither CSF counts (proteins and white blood cells) nor duration of hospitalization differed significantly between these two groups (*P* > 0.05). The abnormal SSRs mainly in patients with AIDP subtype, although the difference between the two groups in electrophysiological subtype (AIDP vs. AMAN) was not statistically significant (*P* = 0.548), but abnormal SSRs occurred mainly in patients with the AIDP subtype. Seven GBS patients (28%) had a poor prognosis 1 month after symptom onset, and the SSR of all these patients was abnormal. All children with GBS had a good prognosis 6 months after onset (Table 3).

**Table 3:** Comparison of clinical data by SSR findings in GBS patients.

**Variable**	**GBS patients with abnormal SSR**	**GBS patients with normal SSR**	**Comparative test statistic**	***P***
Number of patients	18	7		
Age (years), median (IQR)	6 (4–8)	6 (5–8)	*Z* = 0.031	0.976
Male, *n* (%)	9 (50)	5 (71.4)	—	0.407^a^
Preceding event, *n* (%)	14 (77.8)	5 (71.4)		1.000^a^
Time from onset to nadir (days), median (IQR)	4 (3–10)	10 (4–12)	*Z* = 1.739	0.082
Hughes grade at nadir, median (IQR)	4 (2–5)	4 (2–4)	*Z* = 1.052	0.293
**Neurological symptoms**, ***n*** **(%)**				
Facial paralysis	4 (22.2)	2 (28.6)	—	1.000
Bulbar paralysis	6 (33.3)	1 (14.3)	*χ^2^* = 0.208	0.648
Neuropathic pain	12 (66.7)	7 (100)	—	0.137
Autonomic dysfunction, *n* (%)	13 (72.2)	4 (57.1)	—	0.640
Mechanical ventilation, *n* (%)	6 (33.3)	0	—	0.137
**Subtype**, ***n*** **(%)**			—	0.548
AIDP	16 (88.9)	5 (71.4)		
AMAN	2 (11.1)	2 (28.6)		
Protein in CSF (g·L^−1^), median (IQR)	0.92 (0.69–1.69)	0.94 (0.77–1.74)	*Z* = 0.121	0.904
White blood cells in CSF (10^6^/L), edian (IQR)	6 (2.75–11)	4 (2–7)	*Z* = 0.373	0.389
**Treatment**, ***n*** **(%)**				
IVIg	18 (100)	7 (100)	—	—
Plasmapheresis	3 (16.7)	0	—	0.534^a^
Hughes grade at 1 month after onset, median (IQR)	2 (1–4)	1 (1–2)	*Z* = 1.600	0.110
Hughes grade at 6 months after onset, median (IQR)	0 (0–1)	0	*Z* = 0.732	0.464
Short-term prognosis, *n* (%)			*χ^2^* = 2.098	0.148
Good	11 (61.1)	7 (100)		
Poor	7 (38.9)	0		
Duration of hospitalization (days), median (IQR)	23.5 (18–39.3)	24 (18–27)	*Z* = 0.759	0.449

GBS, Guillain–Barré syndrome; AIDP, acute inflammatory demyelinating polyneuropathy; AMAN, acute motor axonal neuropathy; Z, rank sum test.

a Fisher's exact test.

## 4. Discussion

GBS is an autoimmune acute inflammatory peripheral spectrum disease typically characterized by limb weakness with or without sensory disturbance. AIDP and AMAN are the most common subtypes of GBS (17). The long-term prognosis of most patients is considered to be good. However, approximately one-fifth of patients with GBS remain unable to walk independently (18). In this study, 6 (28%) and 18 (72%) of the 25 patients had poor and good short-term prognoses, respectively, while all patients (100%) were able to walk independently at 6-month follow-up, indicating that the long-term prognosis in children with GBS is better than that observed in adults; this can be attributed to the stronger capacity for repair of the peripheral nerves in children.

The sympathetic skin response can be measured via the skin-sweating response to detect the function of post-sympathetic ganglionic C fibers as a reflection of autonomic dysfunction (19). Several studies of SSR in GBS patients have shown that the main findings are the absence of SSR and increased latency or decreased amplitude of the response (7, 20). Most of the previous studies attempted to examine the SSR changes in adult GBS patients rather than pediatric GBS patients. In the present study, pediatric GBS patients showed an absence of SSR and increased latency of SSR, and SSR abnormalities were observed in a higher proportion of patients (72%) compared to the abovementioned studies. In addition, SSR latency in the upper limbs was similar in patients with GBS and in HCs, which could be explained by the fact that eight of the 25 patients with GBS did not elicit waveforms; three of the 17 patients whose data were entered into the statistical analysis showed prolonged onset latency of SSR in the upper limbs, and the sample size for data collected from the upper limbs was relatively small.

The diagnosis of pediatric GBS can be challenging and is highly dependent on electrophysiological studies and cerebrospinal fluid examinations. However, ancillary examinations might produce normal findings in the acute phase of GBS; therefore, there is an urgent need to identify a sensitive and specific marker that can be used not only to identify GBS in the acute phase but also to predict short- and long-term prognosis in clinical practice. A nerve conduction study can be used to assess large myelinated peripheral nerve fibers and is a useful tool for the early diagnosis of GBS in children (21). In this study, there was no difference between SSR and NCS findings in the acute phase of GBS, suggesting that SSR could also be used for the early diagnosis of GBS, especially when NCS is normal in the early phase of GBS. Furthermore, the change in SSR occurred earlier than the change in NCS during the recovery phase, indicating that recovery of autonomic nerve fibers occurred earlier than recovery of peripheral nerve fibers; we speculate that this is related to the neural microstructure. The autonomic nerve is made up of small, thin myelinated nerve fibers, whereas the peripheral nerve is made up of large, myelinated efferent and afferent nerve fibers. Peripheral nerve remyelination and axonal regeneration are natural repair processes, and large nerve fibers need more time to recover. Therefore, SSR could be used as an objective electrophysiological marker for the early diagnosis and follow-up of GBS.

It has been reported that the incidence of AD in children with GBS is approximately 66.7%, and this is mainly characterized by cardiovascular dysfunction (e.g., cardiac arrhythmia or blood pressure instability), gastrointestinal dysfunction (e.g., ileus or constipation), and excessive sweating (4). Consistent with previous studies, 68% of the children with GBS in the present study had GBS complicated by AD, with a higher incidence of hypertension or tachycardia. Given the high clinical risk of autonomic morbidity, identifying AD early in the course of GBS may be important (22, 23). Our study showed that 52% of patients had GBS complicated by AD combined with an abnormal SSR, suggesting that an abnormal SSR response indicates a high risk of AD. Therefore, SSR could be used as a reflection of AD and could constitute an objective electrophysiological marker for the early identification of GBS complicated by AD in children. However, there were four patients with autonomic dysfunction in the absence of an altered SSR response; this could be explained by the fact that only a few autonomic fibers were affected, and the duration of AD was temporary and recovery was spontaneous.

One cohort study of pediatric GBS complicated by AD has shown that AD is significantly associated with increased severity and poor prognosis of GBS (24). In the present study, there was a trend toward correlation between SSR findings and poor clinical motor function, as well as short-term prognosis of GBS. There were 13 (52%) patients with both AD and abnormal SSR findings, among whom five patients underwent mechanical ventilation. Meanwhile, the SSR findings of all patients with a poor short-term prognosis were abnormal. These patients with both AD and abnormal SSR findings had a greater need for mechanical ventilation and more severe motor weakness, as well as experiencing slower overall recovery. Findings on the correlation between SSR and electrophysiological subtype of GBS have been inconsistent across different studies. One study found that an absent SSR indicated demyelinating neuropathy (20), while another has pointed out that SSR abnormalities are associated with axonopathies (25). In our study, SSR abnormalities mainly occurred in cases of the AIDP subtype, and the test was also slightly helpful for differentiating between electrophysiological phenotypes.

The limitations of our study are as follows. First, we mainly analyzed latency of SSR, without considering amplitude. In future studies, our team will analyze the amplitude of SSR and improve the study of SSR in GBS. Second, the sample size was small. Thus, we will enroll more children in the future to better investigate the association between SSR and short- or long-term prognosis in children with GBS.

In conclusion, children with GBS have a higher incidence of AD. Sympathetic skin response is a useful electrophysiological tool for assessment of AD and could be used for the early diagnosis and follow-up of GBS; it may also be slightly helpful in evaluating disease severity and short-term prognosis.

## Data availability statement

The original contributions presented in the study are included in the article/supplementary material, further inquiries can be directed to the corresponding author.

## Ethics statement

This study was approved by the Ethics Committee of the Children's Hospital of Hebei Province. Written informed consent to participate in this study was provided by the participants' legal guardian/next of kin.

## Author contributions

JL and LZ acquired the electrophysiological data. KL and ZZ acquired clinical data and conducted the statistical analysis. MJ designed the experiments, interpreted the results, and drafted the manuscript. SS revised the initial draft and wrote the final version of the manuscript. All authors made a significant contribution to the work and approved the manuscript.

## References

[1] Van den Berg B Walgaard C Drenthen J Fokke C Jacobs BC Van Doorn PA. Guillain-Barré syndrome: pathogenesis, diagnosis, treatment and prognosis. Nat Rev Neurol. (2014) 10:469–82. 10.1038/nrneurol.2014.121 25023340

[2] Zheng P Tian D-C Xiu Y Wang Y Shi F-D. Incidence of Guillain-Barré syndrome (GBS) in China: a national population-based study. Lancet Reg Health West Pac. (2022) 18:100302. 10.1016/j.lanwpc.2021.100302 35024648 PMC8661041

[3] Shahrizaila N Lehmann HC Kuwabara S. Guillain-Barré syndrome. Lancet. (2021) 397:1214–28. 10.1016/S0140-6736(21)00517-1 33647239

[4] Zaeem Z Siddiqi ZA Zochodne DW. Autonomic involvement in Guillain–Barré syndrome: an update. Clin Auton Res. (2019) 29:289–99. 10.1007/s10286-018-0542-y 30019292

[5] López-Hernández JC de Saráchaga AJ Briseño-Godínez ME May-Mas RN Bazán-Rodríguez L León-Manriquez E. Deltoid muscle strength and autonomic dysfunction as independent risk factors for invasive mechanical ventilation in patients with Guillain-Barré syndrome. Int J Neurosci. (2022) 3:1–13. 10.1080/00207454.2022.2082963 35633066

[6] Kondziella D. Autonomic dysfunction in Guillain-Barré syndrome puts patients at risk. Neurocrit Care. (2020) 32:86–7. 10.1007/s12028-019-00793-6 31338746

[7] Yerdelen D Erol T Karatas M. Selective autonomic screening in Guillain-Barré syndrome. Neurol INDIA. (2010) 58:398–402. 10.4103/0028-3886.65926 20644267

[8] Gerawarapong C. Association of peripheral autonomic neuropathy and sympathetic skin response in the patients with diabetic polyneuropathy: a pilot study in Thailand. J Med Assoc Thai. (2015) 98:1222–30. 27004308

[9] Miscio G Pisano F. Sympathetic skin response in amyotrophic lateral sclerosis. J Clin Neurophysiol. (2016) 33:60–5. 10.1097/WNP.0000000000000226 26844971

[10] Aghamollaii V Harirchian MH Modabbernia A Ghaffarpour M Mousavi M Tafakhori A. Sympathetic skin response (SSR) in multiple sclerosis and clinically isolated syndrome: a case-control study. Neurophysiol Clin. (2011) 41:161–71. 10.1016/j.neucli.2011.08.003 22078728

[11] Leonhard SE Mandarakas MR Gondim FAA Bateman K Ferreira MLB Cornblath DR. Diagnosis and management of Guillain-Barré syndrome in ten steps. Nat Re. Neurol. (2019) 15:671–83. 10.1038/s41582-019-0250-9 31541214 PMC6821638

[12] Sejvar JJ Kohl KS Gidudu J Amato A Bakshi N Baxter R. Guillain-Barré syndrome and Fisher syndrome: case definitions and guidelines for collection, analysis, and presentation of immunization safety data. Vaccine. (2011) 29:599–612. 10.1016/j.vaccine.2010.06.003 20600491

[13] Liu S Xiao Z Lou M Ji F Shao B Dai H. Guillain-Barré syndrome in southern China: retrospective analysis of hospitalized patients from 14 provinces in the area south of the Huaihe River. J Neurol Neurosurg Psychiatry. (2018) 3:1–9. 10.1136/jnnp-2017-316930 29326294

[14] Claus D Schondorf R. Sympathetic skin response. The International Federation of Clinical Neurophysiology. Electroencephalogr Clin Neurophysiol Suppl. (1999) 52:277–82. 10590997

[15] Cariga P Catley M Mathias CJ Ellaway PH. Characteristics of habituation of the sympathetic skin response to repeated electrical stimuli in man. Clin Neurophysiol. (2001) 112:1875–80. 10.1016/S1388-2457(01)00647-2 11595146

[16] Hadden RD Cornblath DR Hughes RA Zielasek J Hartung HP Toyka KV. Electrophysiological classification of Guillain-Barré syndrome: clinical associations and outcome. Ann Neurol. (1998) 44:780–88. 10.1002/ana.410440512 9818934

[17] Rajabally YA Uncini A. Outcome and its predictors in Guillain-Barre syndrome. J Neurol Neurosurg Psychiatry. (2012) 83:711–8. 10.1136/jnnp-2011-301882 22566597

[18] Walgaard C Lingsma HF Ruts L. Early recognition of poor prognosis in Guillain-Barre' syndrome. Neurology. (2011) 76:968–75. 10.1212/WNL.0b013e3182104407 21403108 PMC3059137

[19] Lin X Chen C Liu Y Peng Y Chen Z Huang H. Peripheral nerve conduction and sympathetic skin response are reliable methods to detect diabetic cardiac autonomic neuropathy. Front Endocrinol. (2021) 12:709114. 10.3389/fendo.2021.709114 34621241 PMC8490774

[20] Taly AB Arunodaya GR Rao S. Sympathetic skin response in Guillain-Barré syndrome. Clin Auton Res. (1995) 5:215–9. 10.1007/BF01824010 8520217

[21] Rath J Schober B Zulehner G Grisold A Krenn M Cetin H. Nerve conduction studies in Guillain-Barré syndrome: influence of timing and value of repeated measurements. J Neurol Sci. (2021) 420:117267. 10.1016/j.jns.2020.117267 33352506

[22] Chakraborty T Kramer CL Wijdicks EFM Rabinstein AA. Dysautonomia in Guillain-Barré syndrome: prevalence, clinical spectrum, and outcomes. Neurocrit Care. (2020) 32:113–20. 10.1007/s12028-019-00781-w 31297663

[23] van den Berg B Bunschoten C van Doorn PA Jacobs BC. Mortality in Guillain-Barre syndrome. Neurology. (2013) 80:1650–4. 10.1212/WNL.0b013e3182904fcc 23576619

[24] DiMario FJ Edwards C. Autonomic dysfunction in Childhood Guillain-Barre' syndrome. J Child Neurol. (2012) 27:581–6. 10.1177/0883073811420872 22241710

[25] Shahani BT Halperin JJ Boulu P Cohen J. Sympathetic skin response—A method of assessing unmyelinated axon dysfunction in peripheral neuropathies. J Neurol Neurosurg Psychiatr. (1984) 47:536–42. 10.1136/jnnp.47.5.536 6330307 PMC1027833

